# Nanosizing Approach—A Case Study on the Thermal Decomposition of Hydrazine Borane

**DOI:** 10.3390/ma16020867

**Published:** 2023-01-16

**Authors:** Nur Ain Abu Osman, Nor Izzati Nordin, Khai Chen Tan, Nur Aida Hanisa An Hosri, Qijun Pei, Eng Poh Ng, Muhammad Bisyrul Hafi Othman, Mohammad Ismail, Teng He, Yong Shen Chua

**Affiliations:** 1 Hydrogen Energy Storage Research Group, School of Chemical Sciences, Universiti Sains Malaysia (USM), Penang 11800, Malaysia; 2 Dalian Institute of Chemical Physics, Chinese Academy of Sciences, Dalian 116023, China; 3 Energy Storage Research Group, Faculty of Ocean Engineering Technology and Informatics, University Malaysia Terengganu, Kuala Nerus 21030, Malaysia

**Keywords:** hydrogen storage, hydrazine borane, nanosizing

## Abstract

Hydrazine borane (HB) is a chemical hydrogen storage material with high gravimetric hydrogen density of 15.4 wt%, containing both protic and hydridic hydrogen. However, its limitation is the formation of unfavorable gaseous by-products, such as hydrazine (N_2_H_4_) and ammonia (NH_3_), which are poisons to fuel cell catalyst, upon pyrolysis. Previous studies proved that confinement of ammonia borane (AB) greatly improved the dehydrogenation kinetics and thermodynamics. They function by reducing the particle size of AB and establishing bonds between silica functional groups and AB molecules. In current study, we employed the same strategy using MCM-41 and silica aerogel to investigate the effect of nanosizing towards the hydrogen storage properties of HB. Different loading of HB to the porous supports were investigated and optimized. The optimized loading of HB in MCM-41 and silica aerogel was 1:1 and 0.25:1, respectively. Both confined samples demonstrated great suppression of melting induced sample foaming. However, by-products formation was enhanced over dehydrogenation in an open system decomposition owing to the presence of extensive Si-O···BH_3_(HB) coordination that further promote the B-N bond cleavage to release N_2_H_4_. The Si-OH···N(N_2_H_4_) hydrogen bonding may further promote N-N bond cleavage in the resulting N_2_H_4_, facilitating the formation of NH_3_. As temperature increases, the remaining N-N-B oligomeric chains in the porous silica, which are lacking the long-range structure may further undergo intramolecular B-N or N-N cleavage to release substantial amount of N_2_H_4_ or NH_3_. Besides open system decomposition, we also reported a closed system decomposition where complete utilization of the N-H from the released N_2_H_4_ and NH_3_ in the secondary reaction can be achieved, releasing mainly hydrogen upon being heated up to high temperatures. Nanosizing of HB particles via PMMA encapsulation was also attempted. Despite the ester functional group that may favor multiple coordination with HB molecules, these interactions did not impart significant change towards the decomposition of HB selectively towards dehydrogenation.

## 1. Introduction

Over the past 20 years, chemical hydrides have been widely investigated as potential material for hydrogen storage owing to high hydrogen capacity. For instance, ammonia borane and hydrazine borane, which possess hydrogen capacity of 19.6 wt% and 15.4 wt%, respectively, are the few materials that fulfil the material capacity requirement for vehicular hydrogen storage. The former has received significant research attention in the past decade. In contrast, the latter is comparatively less investigated even though its potential as hydrogen storage material has been highlighted since 2009 by Hugle et al. [1] Hydrazine borane consists of a hydrazine (N_2_H_4_) and a borane (BH_3_) group, which are datively bound together via the donation of a lone pair electron from N of N_2_H_4_ to B of BH_3_. Furthermore, the covalent molecule of N_2_H_4_BH_3_ (HB) stabilizes itself in an orthorhombic structure via dihydrogen bonding network between H^δ+^ on N and H^δ−^ on B. In 2011, Sutton et al. [2] reported the regeneration of ammonia borane (AB) spent fuel, which utilized hydrazine as the reducing agent. In that process, HB was formed as an intermediate compound, which was later replaced by NH_3_ to form ammonia borane. From the energy and cost efficiency point of view, a simpler process indicates better efficient energy and cost saving. Therefore, it attracts us to further focus on HB as an alternative to AB for on-board hydrogen storage application.

Thermolytic investigation has showed that HB melts at ~60 °C and starts to decompose over a broad temperature range of 90–140 °C [3]. Similar to AB, dehydrogenation of HB is accompanied by foaming and by-products release, such as N_2_H_4_ and NH_3_, preventing direct use of pristine HB as hydrogen carrier in on-board fuel cell application. To overcome these barriers, tremendous effort has been devoted to improving the hydrogen storage properties of HB. Chemical modification via interaction of HB with alkali metal hydrides have been widely investigated, yielding various alkali metal hydridotrihydroborate, (MN_2_H_3_BH_3_, M = Li, Na, K) [4,5,6,7]. In all MN_2_H_3_BH_3_ reported, higher extent of dehydrogenation can be achieved at lower temperatures, however, traces of N_2_H_4_ and NH_3_ are still detectable. A recent study by Moury et al. [8] showed that the by-products formations from the thermolysis of NaN_2_H_3_BH_3_ can be effectively suppressed by doping NaN_2_H_3_BH_3_ with 5 wt% of NaH, yielding pure hydrogen in the dehydrogenation. In addition, melting oriented dehydrogenation was observed in NaN_2_H_3_BH_3_ and KN_2_H_3_BH_3_, therefore, foaming issue remains a critical challenge. Another strategy to improve the hydrogen storage properties of HB is via catalytic hydrolysis or methanolysis, using transition metals or noble metals as metal catalysts [9,10,11,12,13]. However, the use of solvents (H_2_O or alcohol) makes the hydrogen capacity of the system unattractive for application and the exothermic dehydrogenation indicates the regeneration of spent fuel is of thermodynamically unfavorable. Nanosizing is another approach that has been widely used to improve the reaction kinetic of a hydrogen storage material [14]. In 2005, through quantum-chemical calculations, Wagemans et al. [15] proved that when the crystallite size of MgH_2_ is reduced to below 2 nm, thermodynamic improvement is made possible. Therefore, parallel tuning the thermodynamic and kinetic properties is possible via nanosizing. Several strategies have been employed to control the size of the particle, including mechanically milling, nanoconfinement and polymer encapsulation. Nanoconfinement appears to be the ultimate approach to retain the size of the particles as the growth of particles is constrained by the pore size of the porous materials. In the same year, Gutowska et al. [16] discovered that AB confined within the channel of mesoporous silica (SBA-15) showed significant improvement in the dehydrogenation kinetic and a change in the dehydrogenation thermodynamic as compared to bulk AB. The dehydrogenation temperatures of neat AB at 110 °C and 155 °C were lowered upon confinement. The apparent activation energy for the hydrogen release was lowered significantly from 184 kJ mol^−1^ in the bulk ammonia borane to 67 kJ mol^−1^ in the confined AB. Furthermore, the emission of by-product borazine and the exothermicity of the hydrogen release have been greatly improved as results of the alteration in the decomposition pathway of confined ammonia borane. Lai et al. [17] further investigated the influence of the silica scaffolds on the dehydrogenation from AB/SBA-15 and AB/MCM-41. They found that the confined AB generally had a lower dehydrogenation temperature, released less amount of borazine and diborane but more NH_3_ as compared to bulk AB. Polymer encapsulation is another useful approach in retaining the nanosize of the hydride particle. In 2010, Chen and coworkers [18] utilized PMMA to confine ammonia borane to produce a soft hydrogen storage composite. By using this approach, an air stable composite, which demonstrates rapid dehydrogenation kinetic with high density of hydrogen was obtained.

Thus far, no study on the confinement and polymer encapsulation of HB has been reported. In view of the positive impacts of the nanosizing towards the dehydrogenation of ammonia borane, in this study, we investigated the influence of different nanosizing approaches against the thermal decomposition of HB at different loading. Complete nanoconfinement of HB was achieved by tuning the mass ratio of HB to porous materials (MCM-41 or silica aerogel). In conjunction with the small pore sizes and the presence of Si-O and Si-OH functional groups on the porous framework, the dihydrogen bond network of the confined HB molecules was effectively disrupted and resulted in the absence of the melting process of the HB. The dissociation of B-N bonds was promoted and consequence in the substantial release of N_2_H_4_.

## 2. Materials and Methods

### 2.1. Synthesis of MCM-41

The MCM-41 was synthesized according to procedure modified from Ng et al. [19] A solution was prepared by mixing 6.070 g of cetyltrimethylammonium bromide (CTABr, Merck), 0.700 g of NH_4_OH (25 wt% purity, R&M,) in 60.000 g of distilled water at 80 °C. Next, 22.236 g of sodium silicate (Na_2_SiO_3_, Merck) was mixed with the previous clear solution to give new solution composition of 6 SiO_2_: CTABr: 1.5 Na_2_O: 0.15 (NH_4_)_2_O: 250 H_2_O. The resulting solution was stirred for 15 min before it was hydrothermally treated at 100 °C for 24 h. Then, the pH of the hydrogel was adjusted to 11 by an addition of 25 wt% diluted acetic acid. The hydrothermal process and pH adjustment were repeated twice. The solid product was filtered, washed with distilled water until pH 7, dried overnight at 100 °C and calcined at 550 °C for 10 h to remove the organic template.

### 2.2. Synthesis of Silica Aerogel

A modified method by Rueda et al. [20] was applied in this synthesis of silica aerogel. The synthesis of silica aerogel involved three main steps, which were sol-gel synthesis, aging gel with solvent and removal of organic solvent with supercritical carbon dioxide. Tetraethyl orthosilicate (TEOS, Merck) as a precursor was mixed with absolute ethanol (EtOH, 99.9% purity, Qrec) deionized water and n-hexane (AR grade, Qrec) with a TEOS:EtOH:H_2_O:n-hexane molar ratio of 1:4.4:3.3:4.5. The resulting solution was stirred for 10 min. 0.08 mol of 25 wt% NH_4_OH aqueous solution (R&M) was added as condensation catalyst. Then, the solution was further stirred for 2 h to induce the gelation of silica alcogel particle. The mixture was then left for 2 h to form gel. The formed gel was retrieved and immersed in tetrahydrofuran (THF, Fisher) solvent in a closed vessel to age the gel and strengthen its structure. This process was continued for 7 days, where THF solvent was at least renewed twice to remove traces of ethanol and water. After the aging process, the THF solvent was removed from gel through supercritical CO_2_ drying, which was produced by using CO_2_ premier (99.995% purity, Air Product).

### 2.3. Synthesis of Hydrazine Borane

Hydrazine borane (HB) was synthesized according to the method used by Wu et al. [6]. Approximately 5.000 g of sodium borohydride (NaBH_4_, >96% purity, Sigma Aldrich) was mixed with 100 mL of THF and 12.000 g of hydrazine hemisulphate salt (N_2_H_4_·½ H_2_SO_4_, 98% purity, Sigma Aldrich) was added later in the 500 mL flask. The flask was capped immediately and brought outside the glove box. The flask was then chilled in an ice bath during the first few hours of synthesis as the reaction proceeded vigorously. The flask was connected to a bubbler to release the gas formed during the reaction. This mixture was vigorously stirred and continued for 3 days. Then, the resulting suspension was centrifuged to separate it from the filtrate. The solvent in the filtrate was then removed under reduced pressure to yield white solid product.

### 2.4. Confinement of HB in MCM-41 and Silica Aerogel

In a glovebox, different amount of HB (0.025, 0.050, 0.100 and 0.200 g) were dissolved in 20 mL methanol to form HB methanol solutions. Next, 0.100 g of MCM-41 was added into these solutions to form mixtures with HB:MCM-41 mass ratios of 0.25:1, 0.5:1, 1:1 and 2:1. Methanol was used as solvent due to smaller molecular size of methanol, which can easily diffuse into the small pores of MCM-41. The mixtures were stirred for 2 h and the solvent was removed via reduced pressures, yielding white solid powders. Similar procedure was repeated for confinement of HB in silica aerogel (denoted as Si-Ae hereafter), but with the use of THF as solvent, at HB:Si-Ae mass ratios of 0.25:1, 0.5:1 and 1:1. The final dry solid HB:MCM-41 and HB:Si-Ae were obtained and kept in glovebox.

### 2.5. Encapsulation of Hydrazine Borane with PMMA

The polymethyl methacrylate (PMMA, Mw = 120,000 g mol^−1^, Sigma Aldrich) with different weight were first dissolved with 20 mL of THF. 0.150 g of HB was then added to the solution to form HB:PMMA mass ratios of 0.5:1, 1:1, 2:1 and 4:1. The mixture was then stirred vigorously for 2 h and the solvent was removed via vacuum evaporation.

### 2.6. Characterization

XRD measurements were conducted on a Bruker D8 diffractometer (Cu Kα, 40 kV, 40 mA). A self-made sample cell was used to protect the samples from air contamination. FTIR measurements were conducted on a Perkin Elmer Spectrum 2000 FTIR spectrophotometer. Open system thermal desorption behaviors of the materials were investigated by using a custom-made temperature-programmed desorption-mass spectrometer combined system (TPD-MS) and thermogravimetry and differential thermal analysis (TG-DTA) measurement. In TPD-MS analysis, a sample loading of 10 mg was used and the sample was heated at a heating rate of 5 °C min^−1^ under a dynamic flow mode with purified argon gas as a carrier gas. MS signal of *m/z* ratio of 2 (H_2_), 17 (NH_3_), 28 (N_2_) and 32 (N_2_H_4_) were recorded as a function of temperature. TG-DTA was performed on Perkin Elmer STA8000. A sample loading of ~10 mg was used. The analyses were carried out at different heating rates of 2, 5, 10 and 20 °C min^−1^ in N_2_ atmosphere. Since hydrogen release at high temperatures is not practical for application, the kinetic investigations were carried out by treating the TG-DTA data at different heating rates, from room temperature to 200 °C (which corresponds to the main decomposition step), using Kissinger methods. The closed system decomposition was conducted in a sealed reactor with a total volume of 18.6 mL. A sample loading of 150 mg was used and the sample was heated at a heating rate of 5 °C min^−1^. The released gaseous product was accumulated in the reactor throughout the experiment.

## 3. Results and Discussion

### 3.1. Optimization of HB Loading

#### 3.1.1. DTA Analysis

Although porous material is expected to play important role in tuning the dehydrogenation properties of hydrazine borane upon confinement, it is undeniable that the incorporation of porous material, which does not contribute in hydrogen storage will act as a deadweight that inevitably lowers the hydrogen capacity of the composite materials. Therefore, maximizing the loading of HB in the porous material is important to maintain the attractiveness of the materials for practical application. In this study, TG-DTA was utilized for the optimization of HB loading. Since HB melts prior to the decomposition, the absence of the melting phenomena from DTA is a good indication of the successful confinement of HB as it evidences disruption of intermolecular interaction-dihydrogen bond, which present only in the bulk HB.

**Figure 1:**
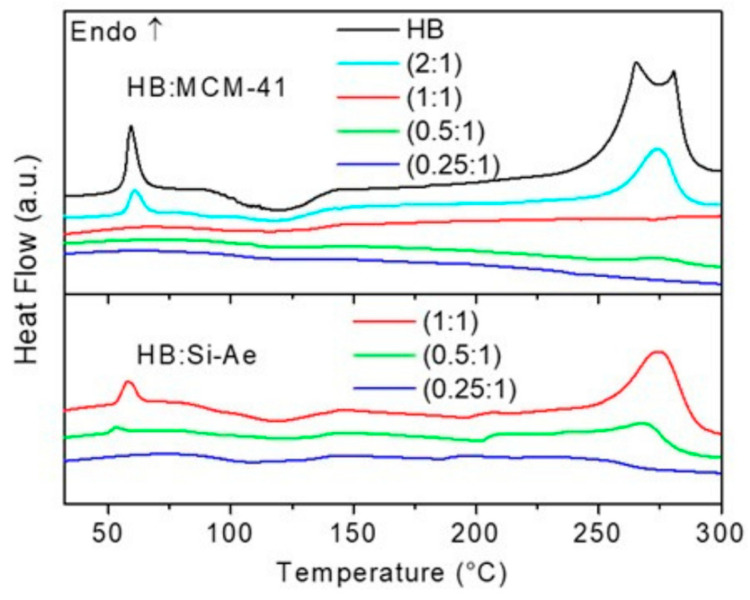
DTA results of HB:MCM-41 and HB:Si-Ae at different loading of HB.

Figure 1 shows the DTA of the MCM-41 and silica aerogel confined HB at different mass ratios. As can be seen, at low loading of HB (0.25:1, 0.5:1 and 1:1 for HB:MCM-41), the HB characteristic melting peak at 60 °C was undetected. These results suggest that all the HB molecules have been successfully infiltrated into the pores of MCM-41. Since the pore size of the MCM-41 was 3.40 nm, the growth of HB particles in the pores is constraint by the pore size, disrupting the intermolecular interactions between HB molecules, thus diminishing the melting process of HB. Furthermore, several exothermic features of HB decomposition in the temperature range of 95–140 °C disappeared and became a broad mild exothermic process upon confinement. These results indicate that nanoconfinement indeed altered the physical and chemical properties of HB. When the loading of HB was increased to 2:1, the melting peak was clearly observed. A possible reason for this observation is that the amount of HB molecules infused into the channels of MCM-41 has exceeded saturation at this loading, which leads to recrystallization and agglomeration of HB on the outside of the MCM-41 that exhibits the properties of bulk HB. Hence, the ratio of 1:1 was determined to be the optimum loading. Similarly in the case of silica aerogel (Figure 1), an optimum ratio of HB:Si-Ae was determined to be 0.25:1.

#### 3.1.2. FTIR and XRD Analyses of HB, MCM-41, Silica Aerogel, HB/MCM-41 and HB/Si-Ae at Various Loading

**Figure 2:**
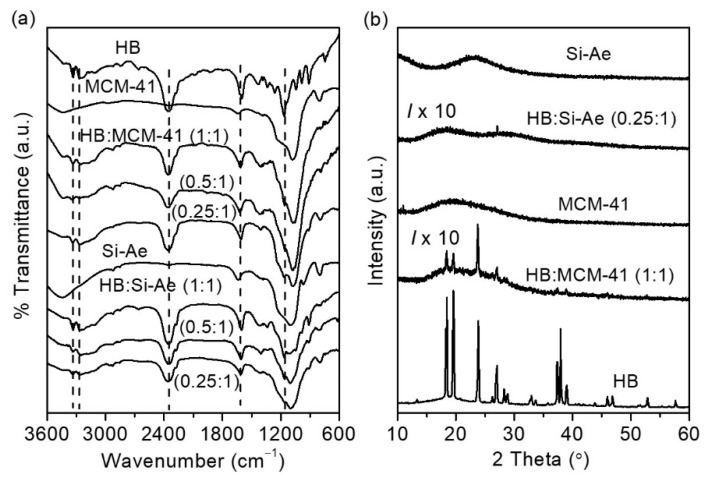
(**a**) FTIR and (**b**) XRD characterizations of HB, MCM-41, silica aerogel, HB/MCM-41 and HB/Si-Ae at various HB loading.

Figure 2a presents the FTIR characterizations of the HB:MCM-41(1:1) and HB:Si-Ae (0.25:1) as compared to bulk HB. The IR spectra of MCM-41 and silica aerogel show typical silica absorptions at ~3430 cm^−1^, which can be assigned to SiO-H stretching, the bands at ~1230 cm^−1^ and ~1080 cm^−1^ are assigned to asymmetric stretching of Si-O-Si groups [19]. The corresponding symmetric stretching modes of Si-O-Si groups are observed at approximately 799 cm^−1^. The IR spectrum of bulk HB shows broad absorbances in the range from 2600–3500 cm^−1^, which can be assigned to multiple stretching of the N-H bonds. The absorbance at 2360 cm^−1^ is attributed to B-H bonds, which is identical to that of ammonia borane [21]. The two overlapping absorbances at 1600 and 1621 cm^−1^ can be ascribed to asymmetric N-H bending and the corresponding symmetric N-H bending can be detected at 1261 cm^−1^ and 1340 cm^−1^, respectively. Other absorbances can also be detected in the fingerprint region, for instances, the broad B-H bending at 1168 cm^−1^, N-H rocking at 1045 cm^−1^ and 984 cm^−1^, BN-N asymmetric at 910 cm^−1^ and B-N stretching at 746 cm^−1^, agreeing with that reported by Moury et al. [3]. Upon confinement, the samples clearly display the features from HB and MCM-41 or Si-Ae, suggesting the HB molecules remained intact within the pores without degradation. XRD characterizations on HB:MCM-41 (1:1) in Figure 2b showed the presence of weak HB diffraction pattern with different relative intensity as compared to the bulk, suggesting the remains of small amount of HB molecules that are arranged in a preferred orientation on the external of MCM-41. XRD of HB:Si-Ae (0.25:1), in contrary, showed complete absence of HB diffraction peak, suggesting a complete infiltration of HB molecules into the pores of silica aerogel.

#### 3.1.3. N_2_ Adsorption/Desorption Isotherm and Morphology Characterizations of MCM-41, Silica Aerogel, HB/MCM-41 (1:1) and HB/Si-Ae (0.25/1)

**Figure 3:**
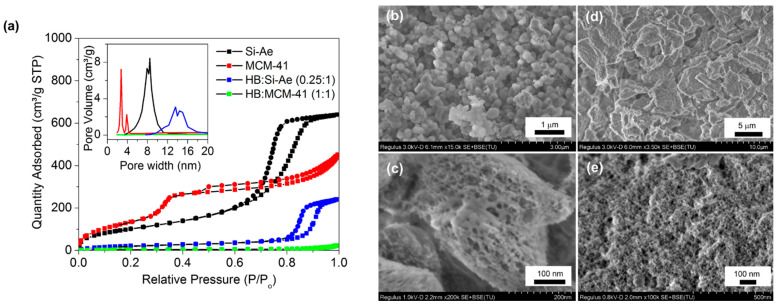
(**a**) Nitrogen adsorption-desorption isotherms and BJH pore size distribution (inset) of MCM-41, silica aerogel, HB:MCM-41 (1:1) and HB:Si-Ae (0.25:1). Solid cubes (■) and spheres (●) denote adsorption and desorption, respectively. (**b**,**c**) SEM images of MCM-41 at 15 k and 200 k magnification, respectively. (**d**,**e**) SEM images of silica aerogel at 3.5 k and 100 k magnification, respectively.

Figure 3a shows the nitrogen adsorption and desorption isotherms of the samples before and after the HB confinement in MCM-41 and silica aerogel. According to the classification of IUPAC, most of the isotherms matched well to class IV isotherm, which is typical for mesoporous material, except for HB:MCM-41 (1:1), which displayed a class II isotherm, indicating a non-porous structure. The hysteresis loop shapes of the mesoporous structures in MCM-41 and silica aerogel are distinctly different. MCM-41 demonstrated a H3 type hysteresis loop at pressure (P/Po) sorption between 0.45 to 0.99 (P is the pressure of N_2_ and Po is the saturation pressure of N_2_), representing the presence of slit-shaped pores at its surface. Silica aerogel showed H1 type hysteresis loop at P/Po sorption between 0.65 to 0.99, suggesting the presence of well-defined cylindrical-like pore channels at their surface. The BET specific surface area and the BJH pore volume of MCM-41 and silica aerogel were estimated to be 639 m^2^ g^−1^, 7.22 cm^3^ g^−1^ and 439 m^2^ g^−1^, 8.48 cm^3^ g^−1^, respectively. Upon confinement, the quantity of gas adsorbed on the materials decreased significantly. The surface area and the free pore volume of HB:MCM-41 (1:1) and HB:Si-Ae (0.25:1) decreased to 10 m^2^ g^−1^, 0.05 cm^3^ g^−1^ and 70 m^2^ g^−1^, 3.08 cm^3^ g^−1^, respectively, suggesting successful infiltration of HB molecules into the pores and even stuffed to fullness in the case of HB:MCM-41 (1:1). SEM micrographs shown in Figure 3b–e clearly illustrated highly porous network of the empty MCM-41 and silica aerogel, respectively.

### 3.2. Thermal Decomposition of HB, HB/MCM-41 and HB/Si-Ae

#### 3.2.1. Open System Decomposition (TPD-MS and TGA)

**Figure 4:**
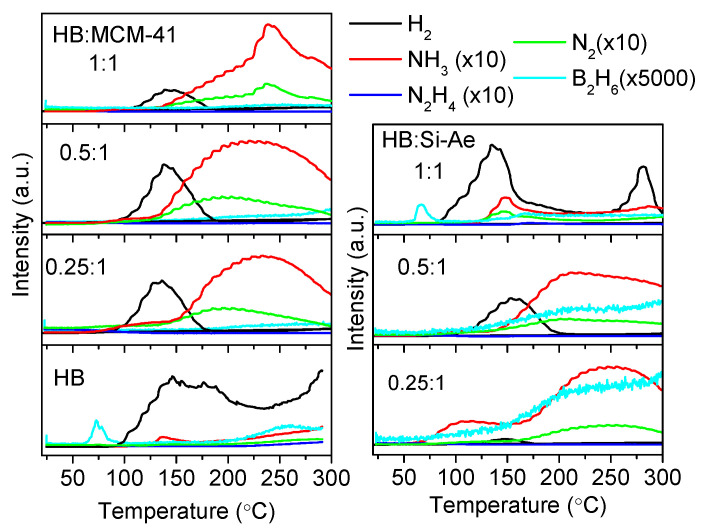
TPD-MS of HB, HB:MCM-41 and HB:Si-Ae at various loading at heating rate of 5 °C min^−1^. For the ease of illustration, the intensity of NH_3_, N_2_H_4_, N_2_ signals in TPD-MS analyses were intensified by a factor of 10, respectively, while B_2_H_6_ signal was intensified by 5000.

**Figure 5:**
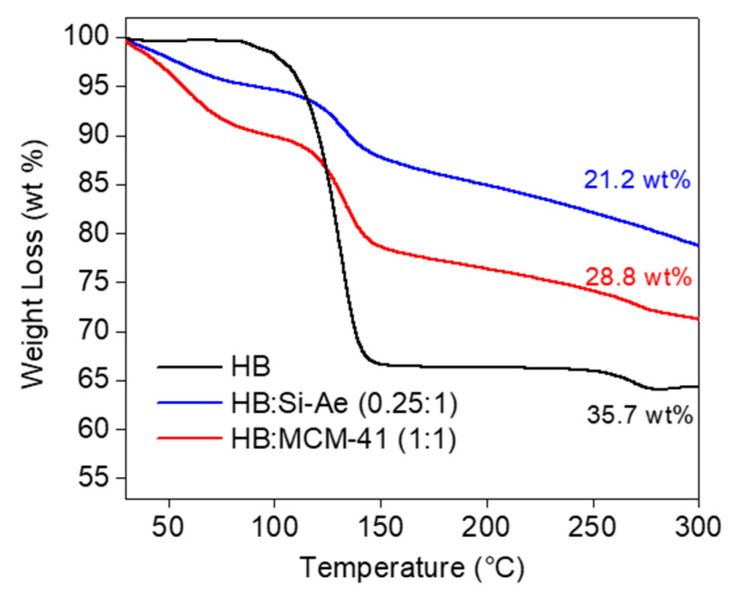
TGA results of HB, HB:MCM-41 (1:1) and HB:Si-Ae (0.25:1) at heating rate of 5 °C min^−1^.

To identify the gaseous species released during the decomposition, the confined samples were subjected for TPD-MS analyses and signal of H_2_ (*m/z* = 2), NH_3_ (*m/z* = 17), N_2_H_4_ (*m/z* = 32), N_2_ (*m/z* = 28) and B_2_H_6_ (*m/z* = 26) were monitored simultaneously. Figure 4 shows the relative intensity of the MS signals of the gaseous products formed from the decomposition of bulk HB, HB loaded in MCM-41 and silica aerogel at various loading, respectively. For bulk HB, small amount of B_2_H_6_ was detected during the melting process at 74 °C. Since no significant weight loss was detected in Figure 5, the release of B_2_H_6_ maybe originated from the side reaction (e.g., decomposition of hydrazine diborane, N_2_H_4_BH_2_(μ-H)BH_3_–a side product formed from the intermolecular interaction between HB molecule and BH_3_ generated from B-N bond cleavage, analogous to ammonia diborane, NH_3_BH_2_(μ-H)BH_3_, that was observed in the decomposition of AB) [22]. H_2_ only began to release at 94 °C and was released primarily up to 300 °C with NH_3_ as the major by-product, which was detected simultaneously at ~100 °C and peaked at 138 °C. Other by-products, such as B_2_H_6_ and N_2_ were present in only a minimum amount at high temperatures. However, N_2_H_4_ was not detected during the decomposition. This may be due to the condensation of N_2_H_4_ on the cold wall of the tubing prior to entering the mass spectrometer. Previous studies had confirmed the release of N_2_H_4_ in the decomposition of HB [3].

For all the confined samples except HB:Si-Ae (1:1), no B_2_H_6_ was detected at 74 °C, indicating that the formation of B_2_H_6_ is melting dependent and the confinement of HB in the porous silica framework impedes the intermolecular interaction to form N_2_H_4_BH_2_(μ-H)BH_3_ intermediate. These samples exhibited a similar dehydrogenation profile where H_2_ was released in stepwise manner. The first step involved rapid dehydrogenation, which peaked at ca. 145 °C and followed subsequently by a sluggish dehydrogenation. This result indicates an identical dehydrogenation mechanism in the confined samples. For the release of NH_3_ and N_2_, a similar trend was detected in MCM-41 and silica aerogel confined samples. In HB:MCM-41 (1:1), NH_3_ and N_2_ were released in a broad temperature range, which began at 130 °C and peaked at 240 °C. As the loading of HB in MCM-41 decreases (HB:MCM-41 (0.5:1) and (0.25:1)), two-step NH_3_ release was detected with the additional step began at temperatures as low as 60 °C. In the case of HB:Si-Ae (0.25:1), which has low loading of HB, a 2-step NH_3_ release profile was also detected, but at much higher relative intensity as compared to H_2_ signal. Therefore, it is plausible to ascribe the release of NH_3_ to a similar deammoniation pathway (i.e., disproportionation of N_2_H_4_), which may be attributed to the higher extent of Si-O···BH_3_(HB) interaction when less HB is encaged in the pores. In additions, the substantial release of NH_3_ suggests that the release of N_2_H_4_ is preferred over dehydrogenation. As the loading of HB in silica aerogel increases (HB:Si-Ae (0.5:1) and (1:1)), the H_2_ release was enhanced. In HB:Si-Ae (1:1), the decomposition profile resembles that of bulk HB. This result is in agreement with the DTA result (Figure 1), which demonstrate the presence of sharp melting peak as results of the presence of excessive HB in the bulk. As can be seen, N_2_ has a similar pattern as that of NH_3_, hence, it is plausible to ascribe the concurrent release of NH_3_ and N_2_ to the disproportionation of N_2_H_4_ [23]. N_2_H_4_ was not detected in all confined samples owing to the possible condensation of N_2_H_4_ vapor during the TPD-MS analyses.

Figure 5 shows the TGA analyses of the optimal loading of HB:MCM-41 (1:1) and HB:Si-Ae (0.25:1) as compared to bulk HB. The decomposition of HB occurred in two steps with a major mass loss detectable in the temperature range of 80–150 °C, achieving 35.7 wt% upon heated to 300 °C. Such a huge weight loss indicates the substantial emission of heavy by-products, i.e., N_2_H_4_ during the decomposition of HB, complementing the results obtained from TPD-MS. In the confined samples, a 3-steps weight loss was detected, achieving 21.2 wt% and 28.5 wt% from HB:Si-Ae (0.25:1) and HB:MCM-41(1:1), respectively, upon heated to 300 °C. The first step of weight loss, which occurred at temperatures < 80 °C corresponds to the detachment of solvent. The second step of weight loss occurred in the temperature range of 80–150 °C to release N_2_H_4_ and NH_3_, similar to that observed in the decomposition of bulk HB. The third step involves a gradual weight loss over the wide temperature range of 150–300 °C, which can be attributed to the predominant release of NH_3_. In both confined samples, their weight losses in the temperature range of 80–300 °C are much lower than that in bulk HB as results of the dead weight contribution from the porous materials. Without considering the dead weight, the weight losses solely contributed by decomposition of HB in HB:MCM-41 (1:1) and HB:Si-Ae (0.25:1) are much beyond the expected value of 35.7 wt% from bulk HB, suggesting substantial release of heavy by-products still remains upon confinement. However, it is also worth to highlight that the excessive weight loss observed in the confined samples may also be contributed by the entrapped solvent, which requires higher temperatures for its complete detachment.

##### Decomposition Kinetics in HB:MCM-41 (1:1) and HB:Si-Ae (0.25:1)

**Figure 6:**
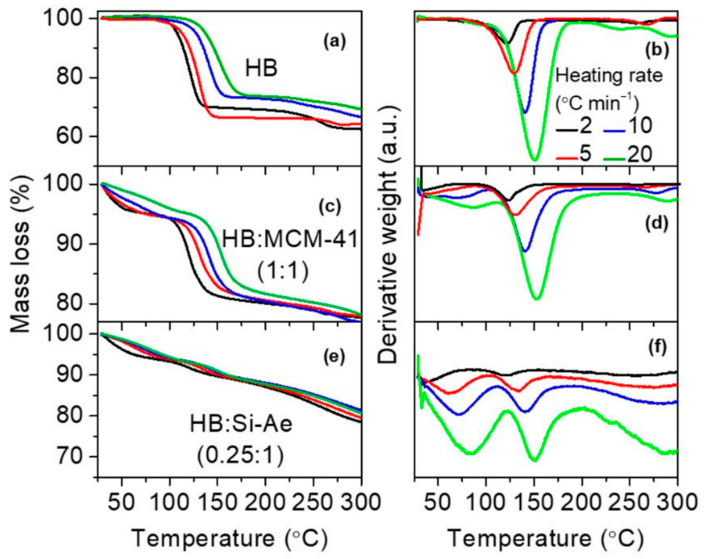
TGA and corresponding DTG of bulk HB (**a**,**b**), HB:MCM-41(1:1) (**c**,**d**) and HB: Si-Ae (0.25:1) (**e**,**f**) at various heating rate heating rates.

**Figure 7:**
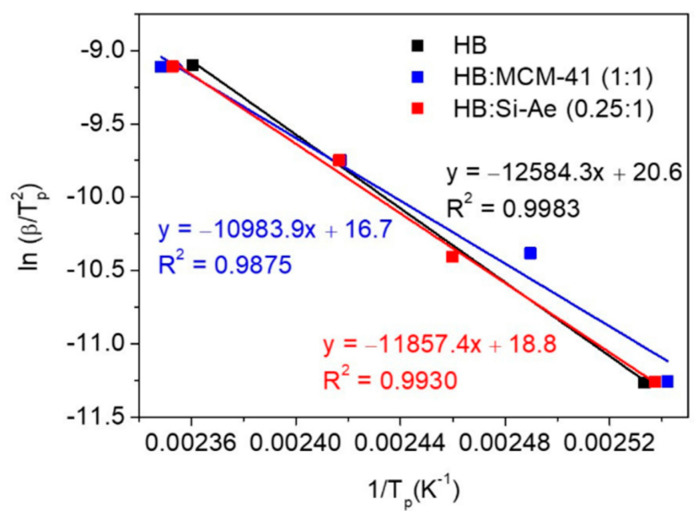
Kissinger plots of HB, HB:MCM-41 (1:1) and HB:Si-Ae (0.25:1) for the main decomposition stage.

The dehydrogenation kinetics of bulk HB, HB:MCM-41 (1:1) and HB:Si-Ae (0.25:1) were investigated by using non-isothermal method for dynamic heating experiments, for instances, TGA, at four heating rates of 2, 5, 10 and 20 °C min^−1^, under nitrogen atmosphere up to 200 °C. The TGA and the corresponding DTG results are shown in Figure 6. The kinetic of the HB decomposition, which corresponds to the main weight loss in the confined samples was investigated by treating the corresponding TGA/DTG results using Kissinger method based on the following equation:

$$ln\left(\frac{\beta }{T_{p}^{2}}\right)=ln\left(\frac{AR}{E_{a}}\right)-\left(\frac{E_{a}}{RT_{p}}\right)$$

where *β* is the heating rate, *T_p_* is the peak temperature, *E_a_* is the activation energy, *A* is the pre-exponential factor and *R* the gas constant. The activation energy (*E_a_*) of the decomposition process can be obtained from the slope of the linear regression. The Kissinger plots shown in Figure 7, demonstrate only minimum change in the activation energy of the main decomposition process, from 104.6 mol^−1^ in bulk HB to 91.3 kJ mol^−1^ in HB:MCM-41 (1:1) and 98.6 kJ mol^−1^ in HB:Si-Ae (0.25:1), respectively, indicating a slightly lower activation barrier upon confinement. These results are somewhat expected since there is no significant change in the decomposition pathway and the main weight losses observed in the confined samples are still mainly attributed to the detachment and decomposition of N_2_H_4_.

#### 3.2.2. Closed System Decomposition (Volumetric Release & FTIR)

**Figure 8:**
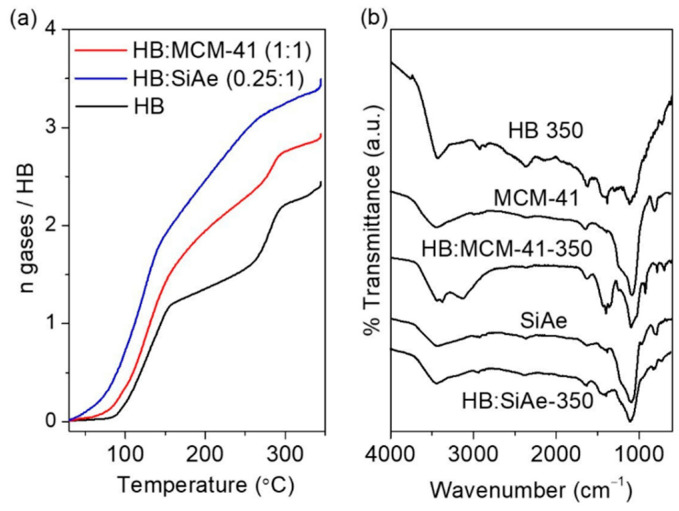
(**a**) Volumetric release measurement of bulk HB, HB:MCM-41 (1:1) and HB: Si-Ae (0.25:1) at a heating rate of 5 °C min^−1^. (**b**) FTIR characterization of the samples collected after decomposition.

Different from the dynamic flow mode (open system) of TG-DTA and TPD-MS measurements, a closed system volumetric release measurement enables the gaseous product to be trapped in a confined space and allow it to further participate in the secondary reaction [24]. Therefore, the decomposition behavior of a closed system may differ compared to that of an open system. In the presence of hydrogen rich B-H and N-H groups under a close system, the H^δ+^ on N_2_H_4_ (or NH_3_) is likely to pair up with all available H^δ−^ on BH_3_ to release H_2_, leaving behind N and B elements in the solid residue as results of the simultaneous B-N bond formation. A closed system volumetric release measurement of HB (Figure 8) showed that decomposition of HB involves two major steps, with the first step begins gradually at ~75 °C, peaked at ~120 °C and followed by gradual release at 150 °C, releasing ~1.5 mole of gaseous product upon heated to 225 °C. The second step followed subsequently and peaked at 261 °C, releasing additional ~0.9 mole of gaseous product upon heated to 350 °C. FTIR characterization on the decomposed HB showed weak absorptions in the range of 2600–3500 cm^−1^ and at 2362 cm^−1^, suggesting that the hydrogen remains in the solid residue appear in the form of N-H and B-H. For HB:MCM-41 (1:1), the gaseous product began to release at lower temperature of ca. 55 °C and liberated 2.3 mole of gaseous product in the first step. Further increasing in temperatures to 350 °C results in the release of additional 0.6 mole of gaseous product, giving a total of 2.9 moles. FTIR characterization on the solid residue showed the remains of weak absorptions correspond to N-H stretching and bending region, suggesting that all B-H has been fully utilized in the dehydrogenation. For HB:Si-Ae (0.25:1), the decomposition started even at 30 °C. As proven by TPD-MS (Figure 4), emission of by-product, such as NH_3_ was detected. Therefore, it is plausible to deduce that the NH_3_ that was released at low temperature may participate in the secondary reaction to release hydrogen. The rate of decomposition in the first step is significantly faster than those of HB:MCM-41 (1:1) and bulk HB owing to a lower barrier reaction involving gas (NH_3_)-solid reaction. Since NH_3_ was also predominantly detected from TPD-MS in the temperature range of 150–225 °C, the improved rate of decomposition observed in the volumetric release under the same temperature range can be unambiguously ascribed to the secondary reaction involving NH_3_. Further increasing the temperature to 350 °C yielded a total of 3.4 mole of gaseous product. The FTIR spectra of the decomposed product resembles to that of silica aerogel, indicating a complete utilization of H atoms in the sample in a close system dehydrogenation since no N-H and B-H bond can be detected in the solid residue.

### 3.3. Decomposition Pathway of HB, HB:MCM-41 (1:1) and HB:Si-Ae (0.25:1)

Decomposition of bulk HB begins with the disruption of intermolecular bonding in the melting process, which is accompanied by the release of B_2_H_6_. However, the release of B_2_H_6_ was not reported in previous study [3]. Its detection suggests possible formation of side product hydrazine diborane during the melting process, which is prone towards decomposition via B-N cleavage to release B_2_H_6_ and N_2_H_4_. In fact, this result agrees with the theoretical calculation reported by Nguyen et al., which proved that the B-N bond cleavage in monomer HB is favored over the hydrogen release to form N_2_H_4_ + BH_3_ (Equation (1)) [25]. The resulting BH_3_ then reacts with the neighboring HB (BH_3_ + HB) to yield hydrazine diborane (N_2_H_4_BH_2_(μ-H)BH_3_) with single bridge B-H-B bond (Equation (2)). However, in the opposite direction of hydrazine diborane formation, B-N cleavage of hydrazine diborane to form B_2_H_6_ + N_2_H_4_ (Equation (3)) is preferred over B-H-B bond cleavage to form BH_3_ + HB (Equation (2)), in agreement with our experimental observation on the detection of B_2_H_6_ during the melting of HB.

N_2_H_4_BH_3_ → N_2_H_4_ + BH_3_, (1)

BH_3_ + N_2_H_4_BH_3_ → N_2_H_4_BH_2_(μ-H)BH_3_, (2)

N_2_H_4_BH_2_(μ-H)BH_3_ ↔ N_2_H_4_ + B_2_H_6_, (3)

Further increasing the temperatures slightly above the melting point of HB results in the release of H_2_, which may involve several pathways derived from HB or HB + BH_3_. As suggested by Moury and coworkers [3], bimolecular interaction of N_2_H_4_BH_3_ to form N_2_H_4_BH_2_N_2_H_3_BH_3_, cyclization of two N_2_H_4_BH_3_ to form N_2_H_3_BH_2_N_2_H_3_BH_2_, propagation of N-N-B oligomers by reacting with unreacted N_2_H_4_BH_3_, dehydrogenation of hydrazine diborane (N_2_H_4_BH_2_(μ-H)BH_3_) via BH_3_-catalyzed H_2_-elimination are possible pathways for dehydrogenation. As the major mass loss of 35.7 wt% was only detectable in the temperature range of 80–150 °C, it is thus plausible to ascribe the mass loss to the release of N_2_H_4_ resulting from B-N bond cleavage of N_2_H_4_BH_2_N_2_H_3_BH_3_. The theoretical mass loss for Equation (4) is 34 wt%, close to that of the experimental value. However, other possible pathways, such as B-N bond cleavages of hydrazine diborane (Equation (3)) and oligomers, which result in the release of N_2_H_4_ are also possible considering myriad of potential structures that may form resulting from N-N-B chains propagation during dehydrogenation.

N_2_H_4_BH_2_N_2_H_3_BH_3_ → N_2_H_4_ + BH_2_N_2_H_3_BH_3_, (4)

As evidenced by TPD-MS (Figure 4), H_2_ was simultaneously released over the broad temperature range from 90 to 300 °C, thenceforth, the remaining B-H rich moieties upon B-N cleavage, i.e., BH_2_N_2_H_3_BH_3_ or BH_3_ may further react with neighboring N-H bearing compounds (i.e., unreacted N_2_H_4_BH_3_, any oligomers or cyclized rings) to release 1 mole of H_2_ simultaneously. Intramolecular interaction between N-H and B-H of the resulting products is also possible to release H_2_ at high temperatures.

**Figure 9:**
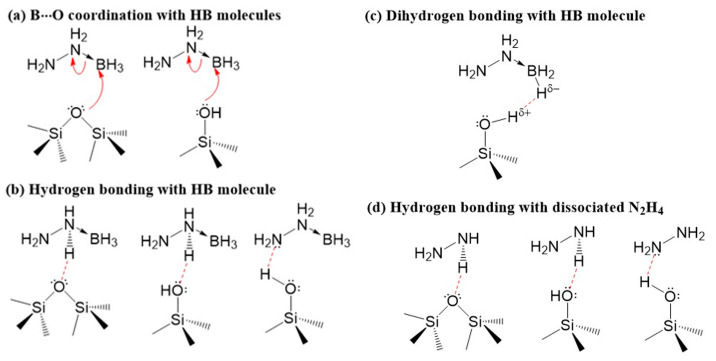
The possible interactions of HB molecules (**a**–**c**) and dissociated N_2_H_4_ (**d**) with surface functional groups (Si-O-Si and Si-OH) in MCM-41 and silica aerogel, respectively.

When HB was confined in the framework of MCM-41 and silica aerogel, the intermolecular interactions were disrupted and the melting induced release of diborane were not detected, suggesting that the formation of hydrazine diborane is unlikely. With the presence of Si-O-Si and Si-OH surface functional groups, formation of multiple interactions with HB molecules (Figure 9a–c) similar to those observed in the confined AB [17], significantly altered the decomposition of HB. The formation of extensive Si-O···BH_3_(HB) coordination (Figure 9a) may further promote the B-N cleavage, making the formation of N_2_H_4_ even more barrierless as compared to that in bulk HB. The resulting N_2_H_4_ is likely to undergo three possible reaction paths: (1) Similar to that of bulk HB, N_2_H_4_ leaches out from the sample as evidenced by the huge weight loss observed in the temperature range of 80–150 °C in TGA. (2) N_2_H_4_ interacts with the surface functional groups of MCM-41 and silica aerogel via hydrogen bonding Si-OH···N(N_2_H_4_) (Figure 9d), facilitating ammonia formation via N-N bond cleavage; (3) The confined N_2_H_4_ may react with the BH_3_ group of the neighboring HB molecules to release H_2_. As NH_3_ was still detectable at temperatures > 150 °C, it is thus plausible to ascribe it to the intramolecular B-N and N-N bond cleavage of the oligomers with short-range structure to release N_2_H_4_ and NH_3_, respectively.

Since MCM-41 and silica aerogel have similar surface functional groups, i.e., -Si-O-Si- and Si-OH, it is thus not surprising to observe similar alteration in the bonding chemistry of HB. However, the difference in the pore size and shape of both porous silica may pose slight change in the decomposition profile of the confined HB. MCM-41 has slit-shaped pores with small pore size, thus it results in smaller HB particles with extensive interaction with the surface functional groups, limiting intermolecular interaction to occur. In contrast, silica aerogel, which has larger cylindrical-like pore channels is likely to permit HB particles to grow inside the channel, enabling more intermolecular interactions to occur and thus demonstrates a different decomposition profile. This is clearly evidenced in the TPD-MS (Figure 4) of the confined HB at low loading of 0.25:1 in which HB:Si-Ae, which was at optimum HB loading released relatively more NH_3_ at temperatures below 150 °C as compared to that in HB:MCM-41, which was underloaded. As significant amount of N_2_H_4_ and NH_3_ were detached during the decomposition process in the dynamic flow mode, (i.e., TPD-MS and TGA analyses), the composition of the product formed are supposed to be N-deficient. However, it is worth mentioning that both NH_3_ and N_2_H_4_ are hydrogen rich compounds and they contribute to hydrogen release via secondary reaction with B-H groups in the sample. In the case when the decomposition process was conducted in the closed system, more H_2_ can be released and N-rich product is likely to form.

### 3.4. Nanosizing via Polymer Encapsulation

**Figure 10:**
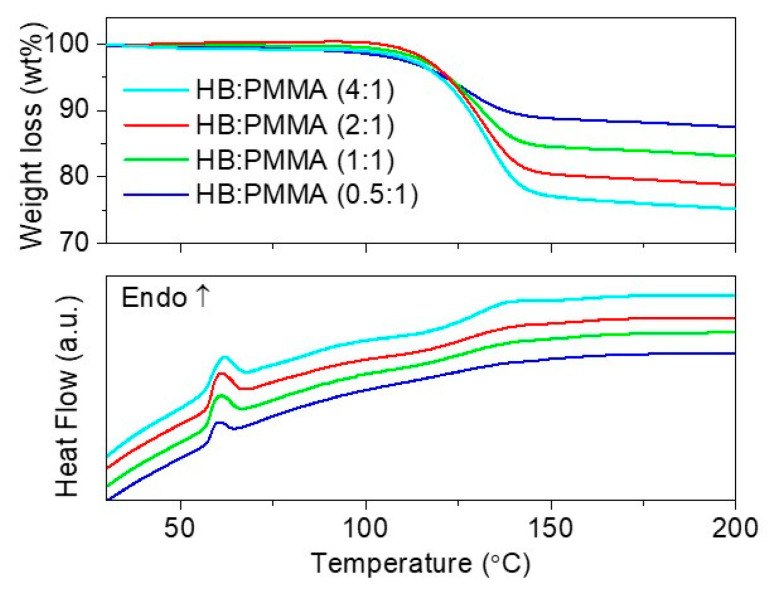
TGA-DTA results of HB:PMMA at different HB loading.

**Figure 11:**
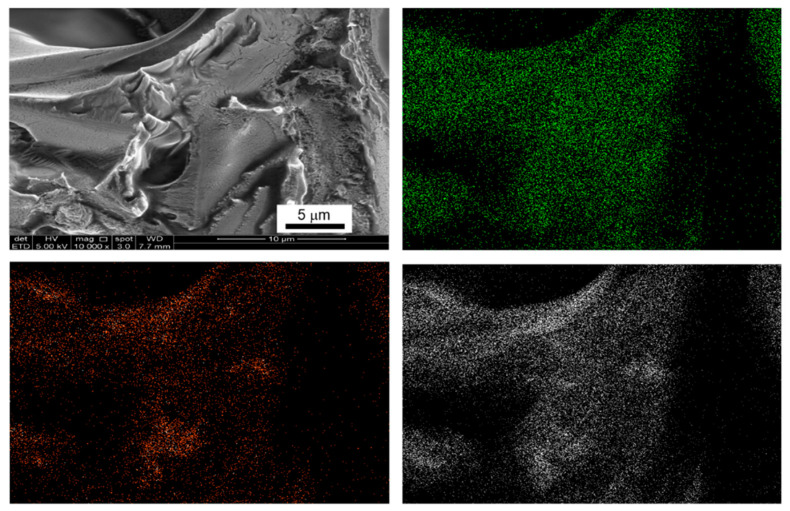
SEM images and EDX mapping of HB: PMMA (0.5:1) (green is referred to boron, orange to nitrogen and white to oxygen).

In view of the fact that polymer encapsulation is also an efficient nanosizing approach [18,26,27], we compared the efficiency of polymethyl methacrylate (PMMA) encapsulation in improving the decomposition of HB with that of nanoconfinement using porous silica. As shown in the DTA analysis (Figure 10), polymer encapsulation of HB did not impart significant change to HB particles as evidenced by the existence of melting phenomena regardless of the HB loading despite good dispersion of HB molecules in PMMA (0.5:1) as shown in SEM-EDX mapping (Figure 11). The main weight loss still occurred in the temperature range of 80–150 °C. Without considering the dead weight contribution and solvent release at temperatures < 80 °C, the weight loss solely contributed by decomposition of HB in HB:PMMA (0.5:1) is ca. 37.5 wt%, close to that observed in bulk HB (Figure 5). This result suggests that PMMA coating, despite with ester functional group that may favor multiple coordination with HB molecules, is equally inefficient in altering the decomposition of HB.

From the results above, it is clearly evidenced that nanosizing of HB particle has been successful via confinement and polymer encapsulation, respectively. However, the decomposition of the nanosized HB particles were enhanced towards by-products formation, suggesting that silicious porous materials and polymer with carbonyl (or ester) group are not appropriate hosts in tailoring the decomposition of HB selectively towards dehydrogenation for hydrogen storage application. To release H_2_ predominantly in the decomposition of HB, a change in the reaction pathway is needed primarily. Therefore, one should consider a host that favors the B-H and/or N-H bond cleavage over the B-N bond cleavage during the decomposition.

## 4. Conclusions

Nanoconfinement is known to be a time-tested approach that has been proven effective in altering both thermodynamic and kinetic properties of hydrides. However, in the case of HB, despite successful infiltration into the pores of porous silica, no significant enhancement in the dehydrogenation selectivity of HB has been observed. The presence of Si-O and Si-OH surface functional groups tends to form multiple interactions with the HB molecules on its surface. Previous calculation has proven that B-N bond cleavage in bulk HB is favored over dehydrogenation. As N_2_H_4_ was still released predominantly in the confined HB, it is therefore believed that the SiO···BH_3_ (HB) coordination makes the B-N bond cleavage even more barrierless. The resulting N_2_H_4_ may interact with the surface functional groups via hydrogen bonding, which promote the formation of NH_3_ via disproportionation. Since N_2_H_4_ and NH_3_ were detached from the sample in an open system decomposition, the solid residue remains in the product are likely to be boron rich. However, in a closed system decomposition, N_2_H_4_ and NH_3_ were retained for the secondary reaction to release more hydrogen, yielding nitrogen rich solid residue. Nanosizing of HB via polymer encapsulation using PMMA was found to be equally inefficient in altering the decomposition of HB selectively towards dehydrogenation as substantial weight loss, which corresponds to the release of N_2_H_4_ remains upon encapsulation.
